# Methylome-Wide Association Studies of Physical Injury Stratified by Depression Status Assesses Exposure by Diagnosis Effects in Oxytocin Signaling and Synaptic Plasticity

**DOI:** 10.1016/j.bpsgos.2026.100710

**Published:** 2026-02-20

**Authors:** Sally Marshall, Rosie M. Walker, Archie Campbell, Caroline Hayward, Andrew M. McIntosh, Kathryn L. Evans, Pippa A. Thomson

**Affiliations:** a Institute of Genetics and Cancer, University of Edinburgh, Edinburgh, United Kingdom; b School of Psychology, University of Exeter, Exeter, United Kingdom; c MRC Human Genetics Unit, Institute of Genetics and Cancer, University of Edinburgh, Edinburgh, United Kingdom; d Centre for Clinical Brain Sciences, Division of Psychiatry, University of Edinburgh, Edinburgh, United Kingdom

**Keywords:** DNA methylation, Oxytocin, Recurrent MDD, Stress response, Traumatic injury

## Abstract

**Background:**

Physical injury is associated with psychological trauma and is a risk factor for major depressive disorder (MDD). DNA methylation has been suggested as a key mediator of psychopathology following trauma.

**Methods:**

We performed phenotypic, methylome-wide association (MWASs) and methylome-wide environment interaction (MWEISs) studies of traumatic injury in individuals with recurrent major depression (rMDD), individuals with single/recurrent MDD (MDD), and control participants (*n* = 4308). We investigated the effect of genetic and epigenetic variation in the biological pathways with evidence of DNA methylation × traumatic injury interactions on the risk of trauma exposure, posttraumatic stress disorder (PTSD), and MDD in an independent sample (*n* = 2759) using gene set enrichment, methylation, and polygenic risk scores, as well as single nucleotide polymorphism (SNP) heritability analyses.

**Results:**

Nominally significant MWEIS association for traumatic injury in control participants versus participants with rMDD was associated with CpG sites at the first exon, 3′ untranslated region, and exon boundaries and in the oxytocin-signaling pathway. Differential DNA methylation in rMDD was associated with gene expression in the limbic lobes and supraoptic nuclei brain regions. Downstream analyses identified pathways associated with rMDD and PTSD including axon development and neuron projection organization for both methylation risk scores and SNP heritability and polygenic risk scores weighted by trauma phenotypes.

**Conclusions:**

Our results suggest that oxytocin and synaptic plasticity–related pathways show differing patterns of DNA methylation associated with traumatic injury dependent on diagnosis, highlighting their involvement in psychopathology after traumatic injury, potentially explaining the heterogeneity seen in response to oxytocin treatment for stress-related disorders. Further studies using longitudinal samples will be required to confirm this.

## Plain Language Summary

Many studies have identified an increased risk of depression after a traumatic event such as a physical injury; however, the biological mechanisms underlying this are unclear. In this study, the researchers examined DNA methylation, an epigenetic mark that can reflect exposure to trauma, and genetic variation and identified oxytocin-signaling and synaptic plasticity–related pathways as potential mechanisms for liability to depression and posttraumatic stress disorder, adding to the evidence that they may be biomarkers for trauma-related disorders.

Traumatic injury is defined as a physical injury that requires urgent medical attention and can be the result of road traffic accidents, falls, violence, or sports injuries. According to the World Health Organization, traumatic injury affects tens of millions of individuals globally each year, resulting in significant long-term disability (1). Approximately 30% to 40% of survivors of traumatic injury develop increasing psychological problems, 9% to 15% of whom develop major depressive disorder (MDD) (2, 3, 4, 5) with elevated rates of depression even in the absence of brain injury (6,7). A history of mental health issues, persistent rumination about the trauma, perceived threat to life, lack of social support, and persistent physical problems are predictors of poor mental health outcomes (8, 9, 10, 11). However, response to treatment of psychological distress is heterogeneous (3,12, 13, 14). Previous studies suggest that epigenetic regulation, such as DNA methylation, may biologically encode the long-term impact of trauma (15, 16, 17, 18), and its study may provide a better understanding of the links between trauma and stress-related disorders.

MDD is a multifactorial disorder with highly polygenic heritability and strong environmental risk factors including adverse life events and trauma exposure. There is evidence that genetic predisposition and adverse life events interact to determine individual risk (19, 20, 21, 22) and that MDD is more heritable in individuals reporting trauma exposure (21). Although trauma is susceptible to recall bias (23), there is evidence that it is associated with a recurrent course of MDD (24, 25, 26, 27). DNA methylation is associated with both self-reported traumatic events and MDD (28, 29, 30) and has been shown to mediate/moderate the effects of trauma on risk on psychiatric illness (31, 32, 33, 34), but the mechanism remains uncertain.

Studying environmental risk factors can be difficult, as they could be a cause or a consequence of MDD. To counteract this issue, some have grouped life events into dependent/independent categories (20, 21, 22, 35, 36, 37), but relatively few studies have looked at external risk factors, such as traumatic physical injury (38). Such injuries can not only be measured objectively, in terms of the injury sustained, but also reflect an individual’s perception of the seriousness of the incident. The perception of trauma may be important for poor mental health outcomes and treatment response (4,39).

We hypothesized that 1) self-reported traumatic injury may be associated with differential methylation and 2) investigating differential methylation in individuals with diagnoses of lifetime MDD and healthy control participants separately may identify the individual differences in response to trauma leading to mental health problems.

**Figure 1:**
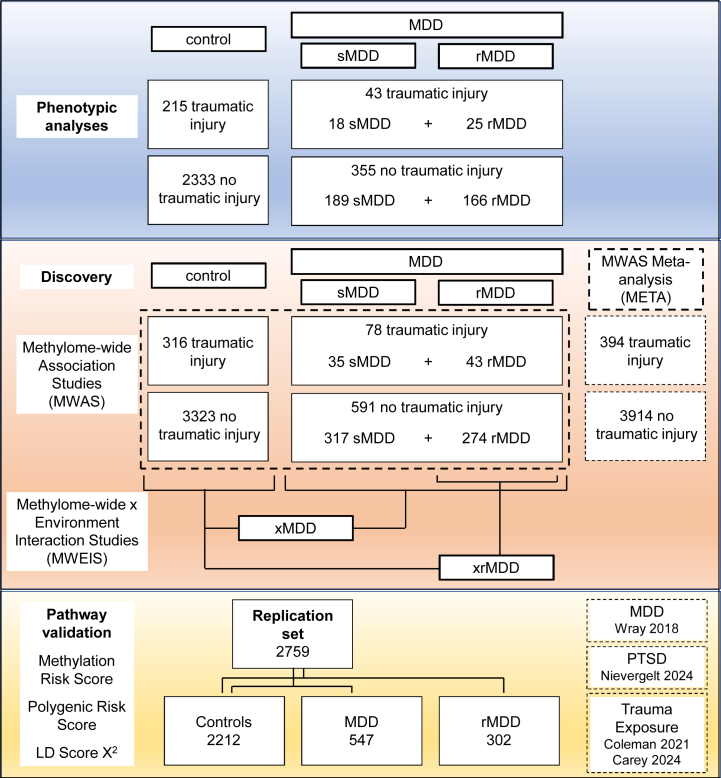
Description of the samples used for the phenotypic, DNA methylation, and validation analyses. LD, linkage disequilibrium; MDD, major depressive disorder; PTSD, posttraumatic stress disorder; rMDD, recurrent MDD; sMDD, single MDD; xMDD, MWEIS of MDD; xrMDD, MWEIS of rMDD.

Methylome-wide association studies (MWASs) were performed to assess differential DNA methylation associated with traumatic injury in 3 groups: a group of participants with MDD, a recurrent MDD (rMDD) subgroup, and a group of individuals without psychiatric diagnoses (Figure 1). We investigated differences in differential DNA methylation between the groups using methylome-wide environment interaction studies (MWEISs) of traumatic injury by diagnosis of MDD (xMDD) and rMDD (xrMDD) and performed an MWAS meta-analysis across all individuals (META). We investigated regional brain expression patterns and biological pathways associated with differentially methylated CpG sites. Finally, pathway-specific methylation risk scores (MRSs), polygenic risk scores (PRSs), and single nucleotide polymorphism (SNP) heritability were used to assess the association of the most significant gene ontology (GO) pathways with trauma exposure, posttraumatic stress disorder (PTSD), and MDD in independent samples (Figure 1).

## Methods and Materials

### Study Cohort

Generation Scotland is a population-based cohort of approximately 24,000 participants (40, 41, 42). Participants were recruited via general medical practices in Scotland between 2006 and 2011 and completed physical test and health questionnaires (data available on request: http://www.generationscotland.co.uk). All participants gave informed consent. Ethics approval for Generation Scotland was given by the National Health Service Tayside committee on research ethics (Reference No. 05/S1401/89).

### Phenotypes

Information on traumatic incidents that resulted in bone fracture was collected as part of the Generation Scotland questionnaire on musculoskeletal history. Participants were asked whether they had ever broken a bone and, if so, whether the fracture had occurred “in a road traffic accident or other serious incident.” In total, approximately 11,800 participants answered that they had broken a bone, 11,387 of whom reported at least 1 fracture. Of those, 1010 individuals responded “yes” to the follow-up question and were classified as having had a traumatic injury.

A diagnosis of MDD was made using the Structured Clinical Interview for DSM-IV (SCID-IV) disorders (43). Participants with diagnoses of schizophrenia or bipolar disorder were excluded from the analyses.

Participants provided blood samples and completed additional questionnaires at baseline (see Supplemental Methods).

### Statistical Analyses

Sample sizes for all steps in the data analyses are provided in Figure 1.

### Phenotypic Analyses

Logistic regression analyses of traumatic injury were performed in R (44) using the *glm* function in MASS (45). Analyses were adjusted for sex and age at baseline. Variables from the Schizotypal Personality Questionnaire-Brief Revised (SPQ), the Mood Disorder Questionnaire, and General Health Questionnaire were log_2_ transformed to improve the normal distribution of the residual variance. Coefficients are reported on the non-log_2_ scale.

### MWASs and MWEISs

Whole-blood DNA methylation was profiled using the Infinium MethylationEPIC BeadChip 850k array (Illumina Inc.), and MWASs were performed as described in Supplemental Methods following Walker *et al.* (46).

Individual MWASs were performed for traumatic injury in 3 groups: control, MDD (single and recurrent cases), and the subset of MDD with evidence for recurrent depression (more than 1 episode of depression noted at the SCID-IV interview). *Limma* was used to calculate empirical Bayes moderated *t* statistics from which the *p* values were obtained (47). The MWAS results for the control and MDD groups were meta-analyzed using an inverse standard error–weighted fixed model implemented in METAL (48) to give the final results set META. See Figure 1 for sample sizes.

MWEISs using differences in differential DNA methylation analyses between the control and MDD groups were performed using a *z* test (49) to test for significant differences in the regression coefficients of traumatic injury between the groups (fold change in the ratio of unmethylated to methylated CpG sites: xMDD and xrMDD). See Supplemental Methods for more details.

A genome-wide significant *p* value threshold (*p*_T_) of <9.42 × 10^−8^ was applied to all MWASs and MWEISs results in the study, following Mansell *et al.* (50).

### CpG/Gene Set Enrichment Analyses

See Supplemental Methods. Briefly, enrichment analyses were performed for functional annotation of CpG sites using Metascape (51). Overlap with drug-associated gene set was tested using Enrichr-KG (52) and the DeepCoverMOA Drug Mechanisms of Action dataset (53). Gene set expression enrichment in adult human brain regions was assessed using hypergeometric tests implemented in ABAEnrichment (54). GO enrichment analyses for each MWAS/MWEIS were performed using WebGestaltR (55) using ranked gene lists.

### MRS Analyses

Standardized unweighted pathway-specific MRSs were calculated from CpG sites with *p*_T_s of .1, .05, or .01 in the xrMDD MWEIS in an independent replication sample of Generation Scotland individuals without traumatic injury information. MRSs were tested for their association with MDD and rMDD. Principal component analyses of the pathway-specific methylation scores were performed using FactoMineR (56) in R to identify principal components of the methylation scores, and the top 5 dimensions were tested for association with rMDD and MDD in the replication sample set with a significance threshold of *p* < .05.

### Analyses of Pathway-Based SNP Heritability

For Generation Scotland genotype details, see Supplemental Methods.

PRSs were calculated using genome-wide summary statistics from genetic analyses of trauma exposure and MDD in the UK Biobank [Coleman *et al.* (21), GCST009982; Carey *et al.* (57), GCST90309343; Wray *et al.* (58); Nievergelt *et al.* (59) using PRSet functions in PRSice-2 (version 2.3.5) (60)] (see the Supplemental Methods). Linkage disequilibrium score regression (61) was implemented to estimate the pathway-specific SNP heritability using the same base summary statistics with a significance threshold of *z* > 4. Enrichment of SNP heritability in pathways, relative to the full base summary statistics, was calculated based on the mean χ^2^ value per SNP, with a significance threshold of *p* < .05.

## Results

### Traumatic Injury Is Associated With Disorganized Thought and rMDD

Traumatic injury was significantly associated with a lifetime diagnosis of depression (*p* = .0048; odds ratio [OR] = 1.67; 95% CI, 1.17 to 2.39), particularly in individuals with evidence of recurrent episodes (OR = 2.10; 95% CI, 1.33 to 3.33; *p* = .0016) but not in those reporting single episodes (OR = 1.31; 95% CI, 0.78 to 2.20; *p* = .30) (Table 1). Individuals reporting traumatic injury had significantly higher scores on the Mood Disorder Questionnaire and the SPQ scale, particularly for the disorganized factor (β = 0.11; 95% CI, 0.05 to 0.18; *p* = .00040).

**Table 1:** Phenotypic Analyses of Traumatic Injury

Variable	Traumatic Injury, *n* = 258	No Traumatic Injury, *n* = 2688	OR/β (95% CI)	*p* Value
Age, Years	50.09 [11.63]	52.23 [12.6]	OR = 1.04 (1.04 to 1.05)	9.0 × 10^−10^∗
Sex, Female	72%	59%	OR = 2.95 (2.26 to 3.84)	1.3 × 10^−15^∗
MDD, MDD/Control	258, 43/215	2548, 355/2333	OR = 1.67 (1.17 to 2.39)	.0048∗
MDD Single Episode, MDD/Control	233, 18/215	2522, 189/2333	OR = 1.31 (0.78 to 2.20)	.3041
MDD Recurrent Episodes, MDD/Control	240, 25/215	2499, 166/2333	OR = 2.10 (1.33 to 3.33)	.0016∗
Digit Symbol Coding	64.97 [16.15]	71.85 [17.64]	β = −2.540 (−4.574 to −0.505)	.0145
GHQ Anxiety and Insomnia^a^	3 (5)	3 (4)	β = 0.110 (0.004 to 0.228)	.0425
GHQ Depression^a^	0 (1)	0 (0)	β = 0.103 (0.014 to 0.200)	.0218
MDQ Total	1 (4.25)	1 (4)	β = 0.200 (0.077 to 0.336)	.0010∗
SPQ Total	3.5 (6)	3 (5)	β = 0.183 (0.062 to 0.316)	.0022∗
SPQ Cognitive-Perceptual	1 (2)	1 (2)	β = 0.142 (0.050 to 0.242)	.0020∗
SPQ Disorganized	0 (1)	0 (1)	β = 0.111 (0.049 to 0.177)	.0004∗

Values are presented as *n*, %, mean [SD], or median (IQR). All analyses fitted age and sex as covariates. The table presents nominally significant findings. Complete details and results are provided in Table S1.

*∗p* Values significant after multiple testing correction for each total score tested (.05/10 = .005).

GHQ, General Health Questionnaire; MDD, major depressive disorder; MDQ, Mood Disorder Questionnaire; SPQ, Schizotypal Personality Questionnaire-Brief Revised.

a GHQ Likert scoring.

### Different Patterns of Differential DNA Methylation in MDD and Control Participants at Functional Sites Associated With Oxytocin Signaling

DNA methylation can alter both gene expression and alternative splicing by differential methylation at specific gene regions (62,63). To investigate this, we compared the mean levels of DNA methylation at CpG sites associated with different functional sequence classes (Table S2).

**Figure 2:**
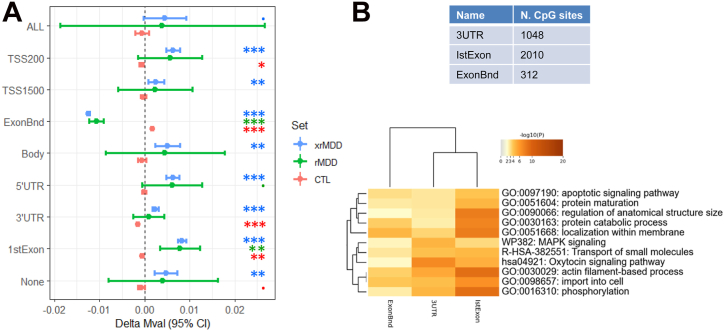
Differences in M value at CpG sites with nominally significant differential methylation (xrMDD *p* < .05) associated with traumatic injury. **(A)** Genome-wide mean differences in CpG M values (delta Mval) by functional annotation. **(B)** GO enrichment for the combined first exon, 3′ UTR, and exon boundary CpG sets. **^.^***p* < .1, ∗*p* < .05, ∗∗*p* < .001, ∗∗∗*p* < .00001. CTL, control; GO, gene ontology; rMDD, recurrent major depressive disorder; TSS, transcription start site; UTR, untranslated region; xrMDD, methylome-wide environment interaction study of traumatic injury by rMDD diagnosis.

Functional annotation was performed for the 40,003 of 772,453 CpG sites that showed nominally significant differences in the patterns of differential methylation in association with traumatic injury in control versus rMDD groups dependent on traumatic injury exposure (xrMDD *p* < .05; 40,003/772,453 = 5.2%). CpG sites in the first exon were underrepresented in those nominally significant for differences in methylation in rMDD versus control groups (xrMDD) (*p* < 1 × 10^−16^) (Table S2A). No other functional class was over- or underrepresented for these sites (Figure 2A and Table S2A). Different patterns of methylation in participants with rMDD and control participants were associated with traumatic injury at sites grouped by functional annotation, particularly at sites associated with the transcription start site (within 200 bp) and 3′ untranslated region (3′ UTR) (control participants only) and the first exon and exon boundaries (control, rMDD, and xrMDD) (Figure 2A and Table S2A).

These results suggest that differential methylation is less likely to occur at first exons, but where it does occur, it is associated with MDD status. Between-group differences in methylation were seen particularly at 3′ UTRs, exon boundaries, and the first exon. This pattern would be consistent with differences in gene expression and/or alternative exon splicing between individuals with or without MDD after traumatic injury (64). This is supported by the identification of components of splicesome complexes in the genes associated with differential methylation patterns at the exon boundaries (*CTNNBL1*, *DDX39B*, *SF3B2*, *SF3B3*, *SRRT*). Protein-protein interaction network analysis of the genes associated with nominally significant CpG sites mapping to the first exon, 3′ UTRs, and exon boundaries indicate enrichment for networks involved in cell population proliferation, synaptic signaling, and vesicle-mediated transport (*p* = 1 × 10^−16^, 1 × 10^−12^, and 1 × 10^−9^, respectively). In the combined gene list of the 3 functional categories, enrichment was seen for both protein-protein interaction networks and GO terms related to the oxytocin-signaling pathway [*p* = 2.5 × 10^−6^ (Table S2A) and *p* = 2.0 × 10^−12^ (Figure 2B and Table S2B), respectively]. Significant enrichment was also found for proteins downregulated by the atypical antipsychotic drug blonanserin (*q* value = .036, overlap = 43/2869 proteins) (Table S2C).

### Little Overlap in CpG Sites Associated With Traumatic Injury in MDD and Control Participants

MWASs of traumatic injury were performed separately in the control, MDD, and rMDD groups (Tables S3–S5). Evidence of differential methylation in the control versus the MDD groups (MWEISs) was also investigated (xMDD and xrMDD) (Tables S6 and S7). Finally, meta-analysis of the control and MDD groups was performed to identify evidence for differential DNA methylation associated with traumatic injury across all individuals (META) (Table S8).

Two CpG sites showed methylome-wide significant differential DNA methylation (*p* value < 9.42 × 10^−8^) in either the MWASs or MWEISs of traumatic injury (Figure S1). CpG site cg14764459 was significant in the MWASs of the control and META groups, and CpG site cg02101279 was significant in the xrMDD MWEIS. Sixty-six CpG sites had *p* values less than a suggestive significance level (*p* < 1 × 10^−5^) (Table S9). Overall, there was little overlap in CpG sites (or genes) with suggestive significance in control participants and participants with rMDD (Figure S2).

### rMDD MWAS Is Enriched for Differential Methylation in the Limbic Lobe and Supraoptic Nuclei

The MWASs/MWEISs were assessed for enrichment for gene expression in adult human brain regions using data from the Allen Brain Atlas (65). Brain regions significantly enriched at a familywise error rate (FWER) <0.05 are shown (Table 2 and Table S10). Three regions were FWER significant in the genes from MWASs of traumatic injury in individuals with rMDD: limbic lobes (MWAS/MWEIS *p*_T_ = .005, No. of genes = 3307, overlap = 1658) and both the left and right supraoptic nucleus (SON) (*p*_T_ = 1 × 10^−5^, No. of genes = 19, overlap = 10 genes). The limbic lobes were also FWER significant in the MWEIS of MDD (xMDD, *p*_T_ = 0.005, No. of genes = 2880, overlap = 1447).

**Table 2:** ABAEnrichment Results of Regional Brain Gene

MWAS/MWEIS	*p*_T_	Allen ID	Structure	FWERs for Expression Quantiles: 0.5, 0.7, 0.9	Minimum FWER
Control	2.3	4219	LL_Limbic_Lobe	.605, .992, .453	.453
MDD	2.3	4219	LL_Limbic_Lobe	.125, .91, .935	.125
rMDD	2.3	4219	LL_Limbic_Lobe	.028, .964, 1	.028
META	2.3	4219	LL_Limbic_Lobe	.855, .944, .727	.727
xMDD	2.3	4219	LL_Limbic_Lobe	.027, .543, .602	.027
xrMDD	2.3	4219	LL_Limbic_Lobe	.595, 1, .983	.595
Control	5	4593	SO_Supraoptic_Nucleus_Left	1, .99, .815	.815
Control	5	4624	SO_Supraoptic_Nucleus_Right	.972, .99, .877	.877
MDD	5	4593	SO_Supraoptic_Nucleus_Left	.660, .992, .96	.660
MDD	5	4624	SO_Supraoptic_Nucleus_Right	.887, .992, .975	.887
rMDD	5	4593	SO_Supraoptic_Nucleus_Left	.034, .3, 1	.034
rMDD	5	4624	SO_Supraoptic_Nucleus_Right	.033, .647, 1	.033
META	5	4593	SO_Supraoptic_Nucleus_Left	1, 1, 1	1
META	5	4624	SO_Supraoptic_Nucleus_Right	1, 1, 1	1
xMDD	5	4593	SO_Supraoptic_Nucleus_Left	.889, 1, 1	.889
xMDD	5	4624	SO_Supraoptic_Nucleus_Right	.984, 1, 1	.984
xrMDD	5	4593	SO_Supraoptic_Nucleus_Left	.616, .997, .985	.616
xrMDD	5	4624	SO_Supraoptic_Nucleus_Right	.581, 1, .995	.581

*p*_T_ indicates −log_10_(*p* values) threshold for gene selection. Complete details are provided in Table S10.

FWER, familywise error rate; MDD, major depressive disorder; MWAS, methylome-wide association study; MWEIS, methylome-wide traumatic injury × diagnosis interaction study; rMDD, recurrent MDD; xrMDD, MWEIS of MDD; xMDD, MWEIS of rMDD.

### Differential Methylation in MDD and Control Participants Shares Gene Ontologies but Not Effect Size

Gene set enrichment analyses, performed on the ranked lists of mapped genes in WebGestalt (55), identified 808 GO terms at a false discovery rate (FDR)–significant *q* value <.05 in at least 1 MWAS/MWEIS of traumatic injury (7.7%) (Figure S5A–D), and 205 of these terms were significant in all 6 (control, MDD, rMDD, xMDD, xrMDD, META) (Table S11).

Comparison of the GO terms found to be enriched at an *q*_FDR_ value <.05 across the MWASs and MWEISs shows that the majority are shared in the analyses of control and rMDD groups (325/563 GO terms for rMDD are shared with the control group) (Figure S3). However, most of these terms are also enriched in xrMDD, suggesting enrichment for differences in effect sizes for the association in control participants versus participants with rMDD (371/563 are FDR significant with a *q* value <.05) (Figure S4).

Normalized enrichment values >2 were found for 18 terms with *q*_FDR_ < .05 (Table S12), 15 of which were FDR significant in all 6 MWASs/MWEISs (hypergeometric *p* = 1.7 × 10^−8^) (Figure S5A). The only cellular component GO term included in this set was that of Schaffer collateral-CA1 synapse (normalized enrichment MDD = 2.04, *q*_FDR_ = .0010). Three terms had normalized enrichment values > 2 in META. These were GABA (gamma-aminobutyric acid) signaling, neuron projection guidance, and mesenchymal cell proliferation (*q*_FDR_ = .0040, .0010, and .0025, respectively).

### Pathways-Specific MRSs Are Associated With MDD Diagnosis

**Figure 3:**
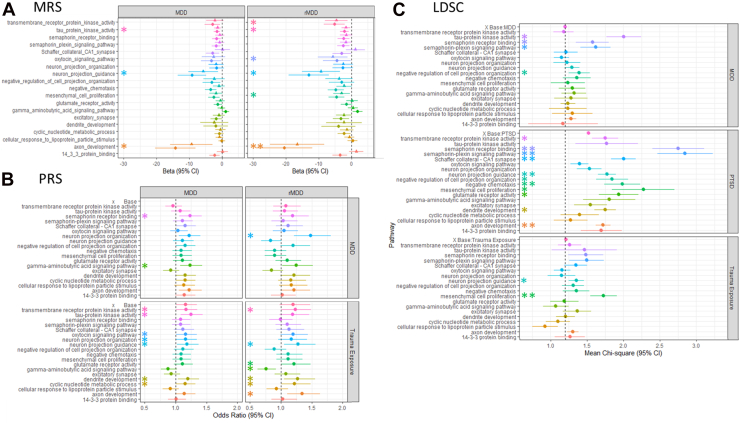
Pathway validation. **(A)** Association of pathway-specific unweighted MRSs with MDD and rMDD in the discovery (circles) and replication (triangles) sets. **(B)** PRS analysis in the Generation Scotland replication sample with weighting from MDD [Wray *et al.* (59)] and trauma exposure [Coleman *et al.* (21)]. **(C)** Pathway-specific LDSC using published genome-wide association study (base): MDD [Wray *et al.* (59)], PTSD [Nievergelt *et al.* (60)], and trauma exposure [Coleman *et al.* (21)], with a vertical dashed line at a mean χ^2^ of 1.2 (mean per single nucleotide polymorphism χ^2^ in the base MDD and trauma exposure analyses) and asterisks representing the *p* value for comparison of the mean χ^2^ of each pathway with the corresponding base set. For full results, see Figure S7. ∗*p* < .05, ∗∗*p* < .001. LDSC, linkage disequilibrium score regression; MDD, major depressive disorder; MRS, methylation risk score; PRS, polygenic risk score; PTSD, posttraumatic stress disorder; rMDD, recurrent MDD.

Nineteen pathways including the oxytocin-signaling pathway and 18 pathways that had normalized enrichment values >2 and *q*_FDR_ value <.05 in any one MWAS/MWEIS analysis were selected for analysis in an independent sample set (Table S12). Unweighted pathway-specific MRSs were calculated using CpG sites with *p* < .1, .05, or .01 in the xrMDD MWEIS and tested for association with MDD and rMDD (Table S13). At the *p*_T_ < .1, 3 pathway-specific MRSs were associated with MDD in both the discovery and replication sets (*p* < .05): axon development, neuron projection guidance, and tau kinase protein activity (*p* ≤ .0048, *p* ≤ .014, and *p* ≤ .013, respectively) (Figure 3A). These pathways were also significant in rMDD (*p* ≤ 8.36 × 10^−5^, *p* ≤ .0017, and *p* ≤ .013, respectively) as well as 3 additional pathways: mesenchymal cell proliferation, transmembrane receptor protein kinase activity, and the oxytocin-signaling pathway (*p* ≤ .018, *p* ≤ .0090, and *p* ≤ .040, respectively) (Figure 3A). There was a strong correlation in effect sizes between the discovery and the replication set (*R*^2^ = 0.85) across all pathways, and the significant effects are all negative, indicating that pathway methylation is protective.

Due to the overlap in genes within the pathways, we performed principal component analyses of the MRSs in both the discovery and replication sets (Figure S6A, B). Dimension 1 was associated with MDD, but not rMDD, in the replication set (MDD *p* = 1 × 10^−2^ and rMDD *p* = .10) (Table S14C and Figure S6C). The strongest contributions to dimension 1 were from the MRSs of axon development, negative chemotaxis, negative regulation of cell projection organization, neuron projection guidance, semaphorin receptor binding, and semaphorin-plexin signaling (Figure S6D).

### Common Genetic Variants Weighted by Trauma Exposure Predict MDD Diagnosis and Are Enriched for SNP Heritability in PTSD

To test for independent evidence for the association of the 19 selected pathways, pathway-specific PRSs weighted by genome-wide association studies (GWASs) of trauma exposure, MDD, or PTSD were tested for association with MDD and rMDD [Coleman *et al.* (21) trauma exposure, Carey *et al.* (57) trauma exposure 2, Wray *et al.* (58) MDD, Nievergelt *et al.* (59) PTSD] (Figure 3B; Table S15 and Figure S7B) and pathway-specific heritability (*h*^2^_SNP_) and enrichment for SNP heritability (χ^2^) investigated within these traits (Figure 3C; Table S16 and Figure S7C).

Weighting PRSs using trauma exposure or PTSD identified more pathways associated with MDD or rMDD diagnosis than weighting by MDD (trauma exposure 11 pathways, MDD 3 pathways, PTSD 7 pathways). Only the PRS for GABAergic signaling was associated with MDD diagnosis when weighted by either trauma exposure or MDD. All 6 pathways with MRSs associated with rMDD in at least 1 weighted PRS analysis (competitive *p* < .05), 5 weighted by trauma exposure, 2 weighted by PTSD, and none weighted by MDD. Similarly, 5 of the 6 pathways associated in the analyses of MRSs were associated with significant SNP heritability (*z* score >4), 3 for trauma exposure, 3 for PTSD, and 2 for MDD. Only oxytocin signaling did not predict significant SNP heritability, although it was nominally significant for PTSD (*h*^2^_SNP_ = 0.048, SE = 0.013, *z* = 3.74, *p* = .00036). Evidence for significant enrichment of SNP heritability per SNP compared with the base GWAS (*p* < .05) was found for 4 pathways in MDD, 3 pathways in trauma, and 11 pathways in PTSD.

The pathway analyses are summarized in Table S17. Overall, these analyses indicate that the pathways identified by MWAS/MWEIS are predictive of MDD diagnosis when weighted by trauma-related traits (trauma exposure and PTSD) rather than MDD and are enriched for SNP heritability, particularly for PTSD.

Full summary statistics for the MWASs and MWEISs can be found in Tables S3–S8. The full results of all other analyses are also provided.

## Discussion

We investigated the association of MDD with self-reported traumatic injury, defined here as bone fracture occurring in a serious incident.

Traumatic injury was associated with a lifetime diagnosis of MDD, particularly of a recurrent course. This is consistent with both the genetic overlap between PTSD and MDD (66,67) and the high genetic correlation between rMDD and PTSD (26). A significant association was also found between traumatic injury and the SPQ cognitive disorganization factor, a measure of deficits in the ability to organize and express thoughts and behavior that is strongly associated with an individual’s psychiatric history and poor mental health after trauma (68, 69, 70, 71), particularly physical abuse and intrusive memories following trauma (72, 73, 74, 75, 76).

Patterns of trauma-associated differential DNA methylation (*p* < .05) in individuals with rMDD versus the control group differed by functional annotation, particularly at CpG sites associated with the first exon, 3′ UTRs, or exon boundaries and enriched for genes involved in oxytocin signaling. Functionally annotated CpG sites associated with first exons are enriched for those more likely to show a moderate/strong correlation between blood and brain (62,63). These results may reflect the association of trauma with a relative reduction in gene expression and/or increased alternative splicing (64,77) in the rMDD group compared with the control group. We also identified enrichment of functionally annotated CpG sites associated with oxytocin signaling and proteins downregulated by blonanserin, a relatively new antipsychotic licensed for use in Asia (78). Blonanserin is associated with hypermethylation of genes involved in axonogenesis, cell adhesion, cell morphogenesis involved in neuron differentiation (79), and response to acute stress (80).

Regional brain enrichment analyses suggested that the genes mapping to CpG sites with differential DNA methylation *p* values <1 × 10^−5^ in individuals with rMDD were enriched for sites in genes expressed in the limbic lobe (Allen Brain Atlas ID: 4219, containing the cingulate gyrus, hippocampal formation, parahippocampus, and piriform cortex) and SON (Allen Brain Atlas ID: 4593, 4624) part of the anterior hypothalamic region. This is consistent with the enrichment of GO term Schaffer collateral-CA1 synapses in all 6 MWAS/MWEIS analyses (*q*_FDR_ < .05). These CA1 neurons receive innervation from local neurons and the amygdala (81), which are important in activity-dependent plasticity (82) and fear extinction (83, 84, 85).

The MWASs/MWEISs suggest that differential methylation associated with traumatic injury is enriched in the same biological pathways in individuals regardless of their mental health diagnoses. Critically, the magnitude and direction of the association may differ at the levels of individual CpG sites, genes, and functional pathways dependent on diagnosis. Pathway-specific MRSs implicated axon development, neuron projection guidance, and tau protein kinase activity as pathways mediating the effects on MDD. These pathways also showed high SNP heritability in previous analyses of 2 trauma exposure GWASs (21,57,86). These results suggest that environmental and genetic modulation of synaptic plasticity may be involved in the between-group differences seen in individuals exposed to traumatic injury. In addition, mesenchymal cell proliferation and oxytocin-signaling MRSs were also associated with rMDD.

Methylation levels of approximately 45% of CpG sites are associated with genotype at SNPs in *cis-* with the gene (83,84). Therefore, we sought to validate the pathways identified from the analyses of DNA methylation using common DNA variants. PRSs and pathway-specific SNP heritability analyses identified associations of trauma-weighted PRSs in key neuronal development and neuronal signaling pathways including axon development, neuron projection guidance, and GABAergic signaling, suggesting possible functional effects on synaptic plasticity (87,88). A GABAergic signaling PRS was also associated with MDD when weighted by MDD and with rMDD when weighted by trauma exposure and has previously been associated with depression (89) and trauma, particularly involving the limbic system (90, 91, 92, 93), and may be important in the suppression of traumatic memories (94). Oxytocin treatment of stress response–associated disorders including anxiety, MDD, and PTSD has been suggested recently [reviewed in (95)]. However, heterogeneity in response to oxytocin administration has been reported in individuals exposed to early-life stress (96) and may be the result of differences in psychopathology between comparison groups and/or prior trauma exposure (97, 98, 99). Our study suggests that DNA methylation in the wider oxytocin network is associated with traumatic injury, and at key functional sequences within the genome, it may be important in response to stress, MDD, and PTSD.

Overall, our results suggest that dysregulation of DNA methylation in oxytocin and GABAergic signaling and of synaptic plasticity in the limbic lobes and SON are associated with traumatic injury. Significant pathway-specific per-SNP heritability was identified compared with the full base models in MDD and trauma exposure, but particularly in the analysis of PTSD. This suggests that the effects on synaptic plasticity may be derived from both DNA methylation differences and common genetic variation. These brain regions and processes are known to be involved in stress response (86) and fear memory encoding/extinction (97,99, 100, 101, 102).

This study has limitations, including the fact that the self-report of fracture in a serious accident is a retrospective assessment subject to recall bias. We did not assess brain injury, which may further increase the prevalence of psychiatric illness (86). DNA methylation was measured in whole blood, improving the available sample size and biomarker identification but likely reducing the power to detect regional or cell type–specific associations (103). Furthermore, we were unable to examine the temporal association of the phenotypes. Therefore, differences in DNA methylation levels could reflect genetic susceptibility loci, disease state, or medication. The collection of longitudinal samples will be required to distinguish between potential causal mechanisms. Finally, the trauma-weighted PRS used published adult trauma GWASs, but these phenotypes may differ from the traumatic physical injury that we used (35).

### Conclusions

This study suggests that traumatic injury is associated with widespread differential DNA methylation enriched in synaptic plasticity, oxytocin, and GABAergic signaling pathways and, in combination with common genetic variants in the GABAergic signaling pathway, mediates the risk of MDD. While these results add to the evidence that oxytocin signaling may be a biomarker for trauma-related MDD and response to oxytocin treatment, these results require replication in larger cohorts and the use of longitudinal study designs. Such studies may help identify biomarkers for the long-term effects of traumatic injury on mental health and suggest therapeutic approaches.

## Supplementary Material

Tables S1–S17

Key Resources Table
