# Supplementary material for: Methylome-Wide Association Studies of Physical Injury Stratified by Depression Status Assesses Exposure by Diagnosis Effects in Oxytocin Signaling and Synaptic Plasticity
Source: Biol Psychiatry Glob Open Sci. 2026 Feb 20;6(3):100710. doi: 10.1016/j.bpsgos.2026.100710 (PMC13094437; doi:10.1016/j.bpsgos.2026.100710)
Supplement: Supplemental Methods and Figures S1–S7 [file mmc1.pdf]

SUPPLEMENTARY INFORMATION

Methylome-Wide Association Studies of Physical Injury Stratified by Depression Status Assesses Exposure by Diagnosis Effects in Oxytocin Signaling and Synaptic Plasticity

Marshall *et al.*

Supplementary Methods

Generation Scotland additional phenotypic data.

Participants completed additional questionnaires at baseline.to assess cognitive ability (1-3) and personality traits (4) and symptoms associated with depression (General Health Questionnaire – 28, GHQ) (5), mood disorder (Mood disorder Questionnaire Likert score, MDQ)(6) and schizotypy (Schizotypal Personality Questionnaire sum score – Brief Revised, SPQ-BR)(7, 8). GHQ questions were scored on the Likert scale (0 = “Better than usual”, 1 = “Same as usual”, 2 = “Worse than usual”, 3 = “Much worse than usual”) and summed for all 28 questions and the symptom subscales. MDQ and SPQ symptom scores were calculated as a sum of the individual questions (0 = “No”, 1 = “Yes”).

MWAS & MWEIS

Whole blood DNA methylation was profiled using the Infinium MethylationEPIC BeadChip 850k array (Illumina Inc.) in two sets of GS participants at two separate times by the Wellcome Trust Clinical Research Facility, Edinburgh. The natural discovery sample comprised 5190 individuals and the natural replication sample included 4583 individuals, as described previously (9-11). The two samples were normalised, and the data was converted to M-values. Individuals in the replication sample were unrelated to those in the discovery set (SNP-based relatedness < 0.05). The data was quality controlled prior to the analyses to remove poor-performing probes, sex chromosome probes, individuals with unreliable self-report data, suspected XXY genotype, as described in Walker et al.60 Covariates were fitted to the discovery and replication sample following Walker *et al.* (12). The discovery sample M-values were pre-corrected for relatedness, estimated cell count proportions (granulocytes, natural killer cells, B lymphocytes, CD4+ T lymphocytes, and CD8+ T lymphocytes) and processing batch. This sample was also corrected for age, sex, smoking status, pack years, 20 methylation PCs and 20 genetic PCs. The replication sample M-values were corrected for age, sex, smoking status, pack years, estimated cell count proportions, processing batch, 20 methylation principal components and 20 genetic principal components.

Individual MWAS from each of the two initial samples were performed for traumatic injury in three groups: controls, MDD (single and recurrent cases) and the subset of MDD with evidence for recurrent depression (more than one episode of depression noted at SCID interview, rMDD). Limma was used to calculate empirical Bayes moderated t-statistics from which the *p-v*alues were obtained (13). The discovery and replication sets were meta-analysed using an inverse standard error-weighted fixed model implemented in METAL,[62] only the meta-analysis results are reported. The MWAS results for controls and MDD were meta-analysed using an inverse standard error-weighted fixed model implemented in METAL to give the final results set META.

The final sample sizes for the methylation analyses were: MDD (single or recurrent) n = 669 (78 with reported traumatic physical injury, 591 without), recurrent MDD n = 317 (43; 274); and controls n = 3,639 (316; 3,323). The MDD and control meta-analysis included 772,453 CpG sites from 4,308 individuals (394 MDD; 3,914 controls), Supplementary Table 1.

MWEIS were performed using a Z-test (14) to test for significant differences in the regression coefficients of traumatic injury between the groups (fold-change in the ratio of unmethylated to methylated CpG sites) of the association with traumatic injury in the control versus MDD groups to give the datasets xMDD and xrMDD.

A genome-wide significant *p-v*alue threshold, *p-v*alue < 9.42×10^{-8}, was applied to all MWAS and MWEIS results within the study, following Mansell *et al.* (15).

Gene set enrichment analyses

CpG sites were mapped to genes based on nearest transcriptional start site within 10 Kb. Genes associated with CpG sites filtered by functional annotation and a *p-v*alue < 0.05 in xrMDD were analysed using Metascape (16) Overlap with drug associated gene set was tested using Enrichr-KG (17) and the DeepCoverMOA Drug Mechanisms of Action dataset (18). Gene set expression enrichment in adult human brain regions was assessed using hypergeometric tests implemented in ABAEnrichment65 using genes mapping to CpG sites at three MWAS *p-v*alue thresholds (T1 $p < 2.668 \times 10^{-6}$, -logP = 5.5738; T2 p < 1 x 10-5, -logP = 5; T3 $p < 0.005$, -logP = 2.3) and three expression quantiles: 0.5, 0.7, and 0.9. Brain regions were considered significant at a family-wise error rate (FWER) < 0.05.

Gene set enrichment analyses for gene ontology terms for each MWAS/MWEIS were performed using WebGestaltR (19) using ranked gene lists. Genes were ranked by the most significant CpG site +/- 10kb (-log10(*p*)), no p-value threshold was applied. Analysis of ranked gene lists provides estimates for enrichment for genes within the gene set among the most significant genes (enrichment scores) and normalised enrichment scores that provide a permutation correction (1,000 permutations) for the size of the gene set, so that scores can be compared across sets. The resulting *p*-value is for the significance of the enrichment score compared to the permutations and an FDR correction is applied to adjust for the number of gene sets tested. Gene ontologies were considered significant at an FDR q-value < 0.05 where the overlap contained a minimum of five genes in the target list.

Methylation risk score (MRS) analyses

Standardised unweighted pathway-specific methylation risk scores were calculated from CpG sites with $p < 0.05$ in the rMDD XWAS in an independent replication sample of Generation Scotland individuals without traumatic injury information (MDD (single or recurrent) n = 547, recurrent MDD n = 302; and controls n = 2,212). This set of individuals did not include, and were unrelated to, those individuals in the MWAS/MWEIS. Methylation levels in this sample were measured in the same batches as the discovery set, but covariate adjustment was performed independently. The number of CpG sites varied across p-value thresholds and pathways (Supplementary Table 14). Once calculated scores were tested for association with MDD and rMDD adjusted for age and sex. Principal component analysis of the pathway-specific methylation scores was performed to identify principal components of the methylation scores using the R package FactoMineR (20) and the top 5 dimensions tested for association with rMDD and MDD in the replication sample set.

Analyses of pathway-based SNP heritability

Polygenic risk scores (PRS) analyses were performed in the replication set (see MRS above). Generation Scotland genome-wide genotype data were generated using the Illumina HumanOmniExpressExome-8 v1.0 DNA Analysis BeadChip (San Diego, CA, USA) and Infinium chemistry by the Clinical Research Facility, University of Edinburgh. Full details can be found: (21, 22).

Polygenic risk scores were calculated using genome-wide summary statistics from genetic analyses of trauma exposure, PTSD and MDD in UK Biobank (Coleman et al, 2021, GCST009982; Carey et al 2024, GCST90309343; Nievergelt et al., 2024, Wray et al 2018) (23-26) using PRSet functions in PRSice-2 (v2.3.5) (27) to calculate genome-wide and pathway specific PRS. Independent SNPs were selected using clumping with a window of 250 kb, threshold *p-v*alue = 1 and an r2 = 0.1. PRS were derived for six *p*-value thresholds (5×10^{-8}, 1×10^{-5}, 0.001, 0.05, 0.5, 1). Gene regions were defined using hg19 with 2 kb flanking regions 5' and 3' of the transcription start and stop sites. All PRS were standardised to a mean of zero and a standard deviation of 1. Significance was accepted at $p < 0.05$ using the competitive *p-v*alue derived after 10,000 set permutations. The competitive permutations test for enrichment of signals in the tested pathways compared to match numbers of post-clump SNPs selected from the whole genome (Base set). Analyses were corrected for age, sex and 10 genetic PCs.

LDSC (28) was implemented to estimate the pathway specific SNP heritability of trauma exposure and MDD using the GWAS summary statistics from Coleman *et al.* 2021, Carey *et al.* 2024, Nievergelt et al., 2024 and Wray *et al.* 2018 (excluding Generation Scotland) (23-26). Enrichment of SNP heritability in pathways, relative to the full summary statistics, was calculated based on the mean chi-square value per SNP.

Supplementary Figures

Supplementary Figure 1: Manhattan Plots for MWAS and MWEIS

X-axis genome position of CpG sites by chromosome, Y-axis -log10(*p-v*alue). Methylome-wide significant CpG sites (*p-v*alue < 9.42 x 10^{-8}) are labelled. Methylome-wide significant differential DNA methylation associated with traumatic injury was identified at two CpG sites: cg14764459 (Control *p* = 5.1 x 10^{-8}, META *p* = 6.8 x 10^{-8}) and cg02101279 (rMDD *p* = 8.6 x 10^{-8}). Differential DNA methylation at CpG site cg14764459 is associated with vannin-2 (VNN2) (29) and C-reactive protein (CRP) levels, and type 2 diabetes (30, 31). CpG site cg02101279 maps to a lncRNA transcript, ENSG00000227880. Given the small sample sizes used, these associations require replication.

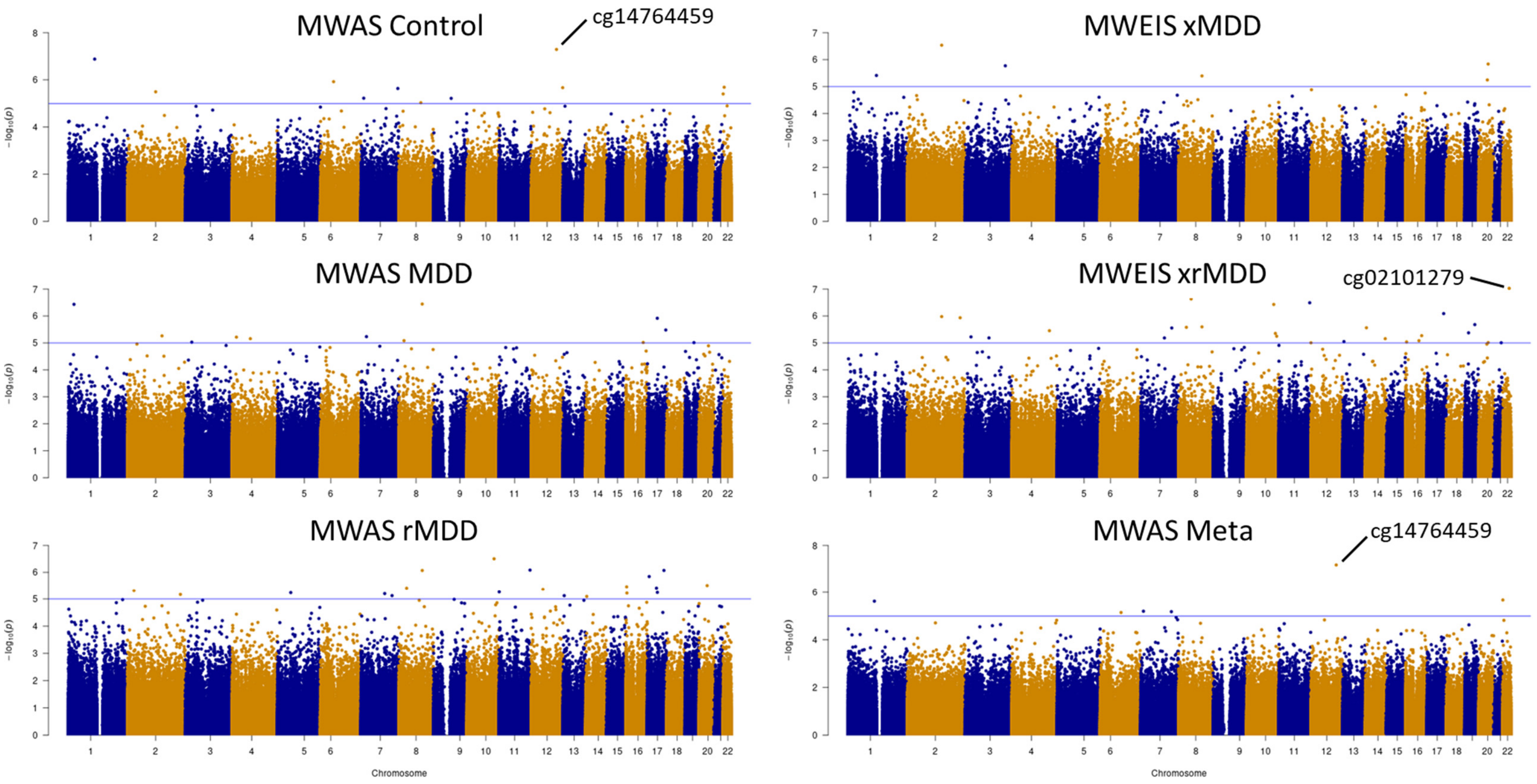

Supplementary Figure 2: Overlap of genes mapped to CpG sites with *p* < 1 x 10^{-5}

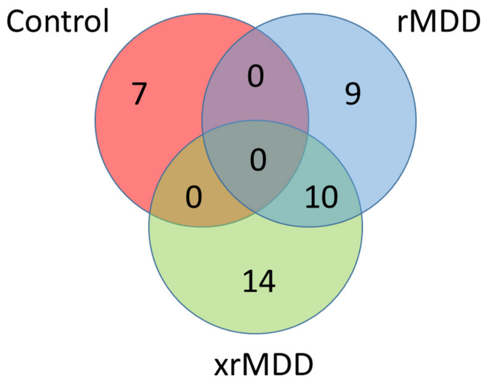

Supplementary Figure 3: Overlap of Gene Ontology terms (FDR q < 0.05) between MDD, rMDD, control & META

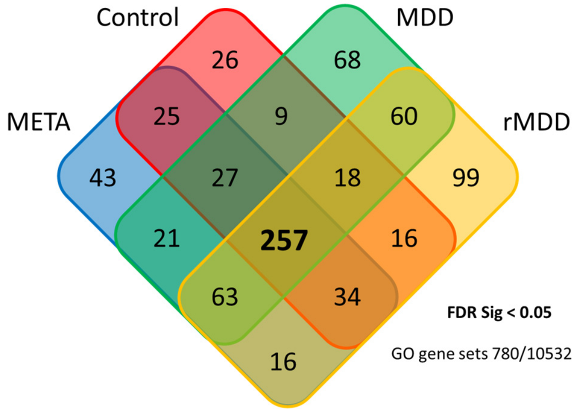

Supplementary Figure 4: Overlap of Gene Ontology terms (FDR q < 0.05) between xMDD, xrMDD, control & META

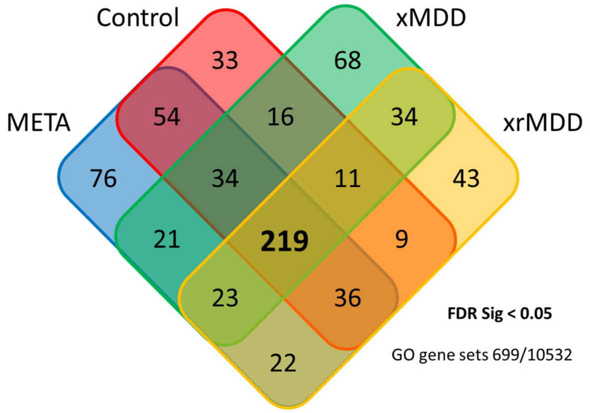

Supplementary Figure 5 Gene Ontology enrichment

Gene set enrichment analyses of MWAS/MWEIS showing a) the overlap of gene ontology terms (FDR < 0.05) between MWAS. b) 18 gene ontologies enriched for evidence of differential DNA methylation with FDR q-value < 0.05 and a normalised enrichment scores of > 2 in at least one group. c) 9 gene ontologies enriched in all groups and showing not significant in either MWEIS. d) 22 gene ontologies significantly enriched in the MDD MWAS and MWEIS analyses but not in the control or META. Bubble plot point size represents the normalised enrichment score (NEScore) and the point colour represents the minus log10 of the FDR q-value. The dendrogram shows the clustering of the gene ontologies based on the NEScores using the control, MDD and rMDD enrichment values.

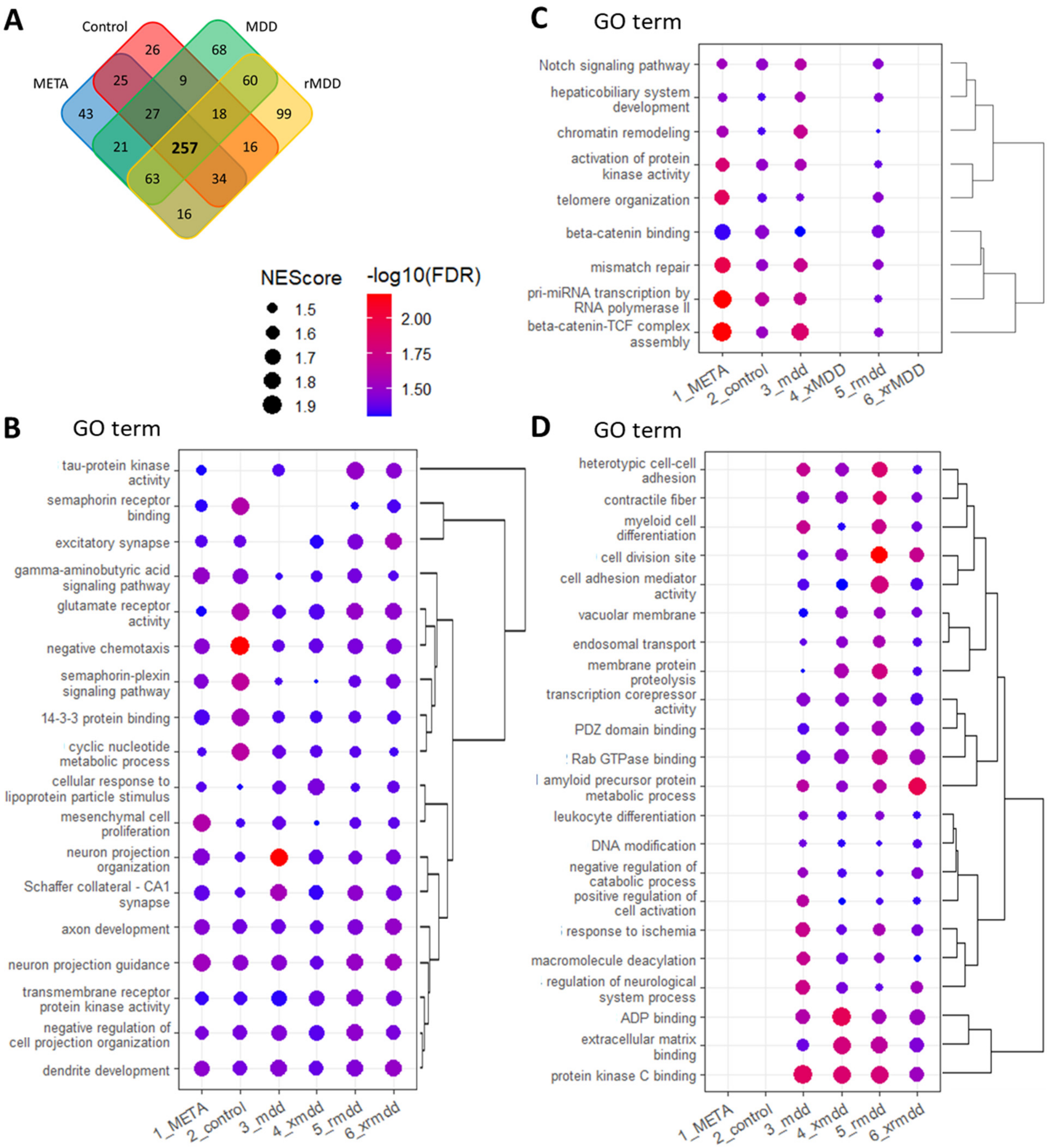

Supplementary Figure 6 MRS Principal Component Analysis of the discovery and replications sets

a) Factor Maps showing the importance of each pathway (cos2), b) the percentage of variation explained by the top ten principal components (eigenvalues), c) association of the top five principal components with diagnosis, d) contributions of each pathway to the top five principal components. *p-v*alues: . < 0.1, * < 0.05, **<0.001

A

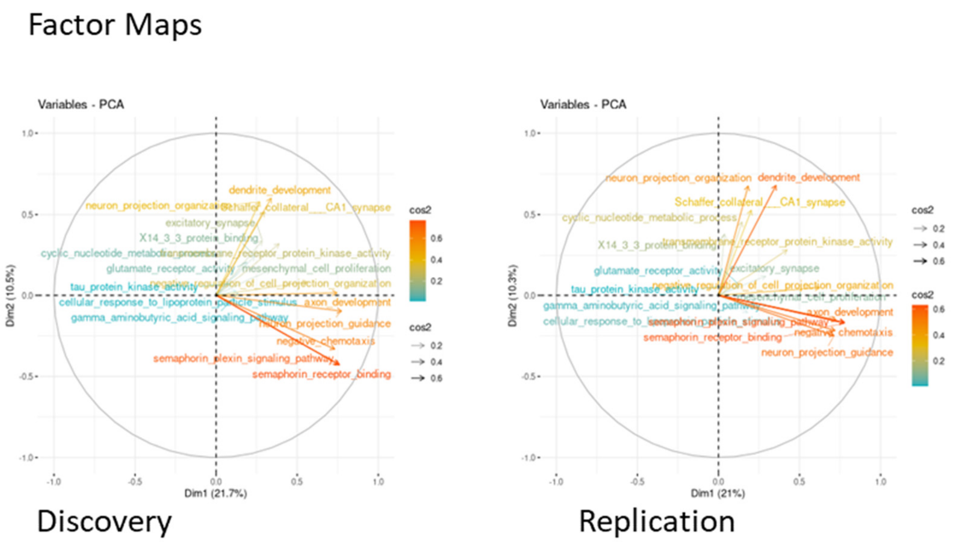

B

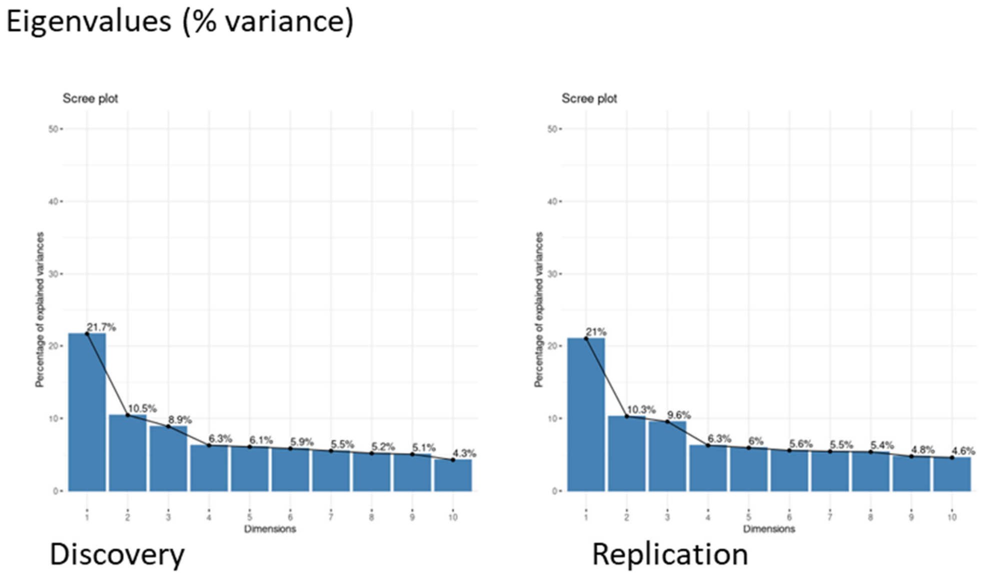

C Association with MDD and rMDD

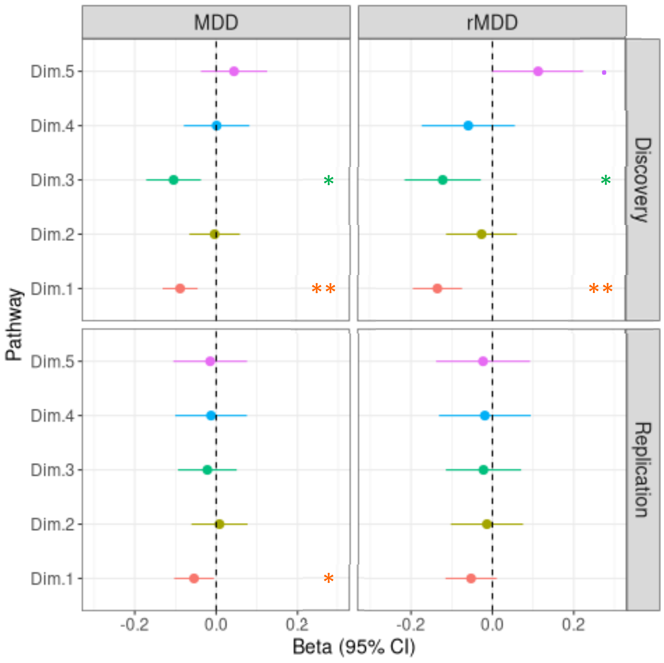

D

Contributions

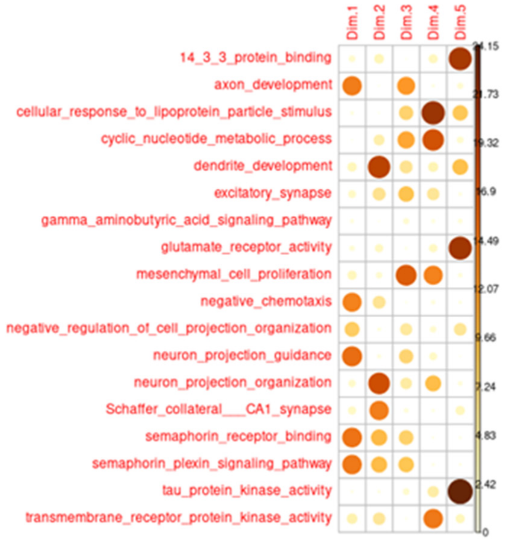

Discovery

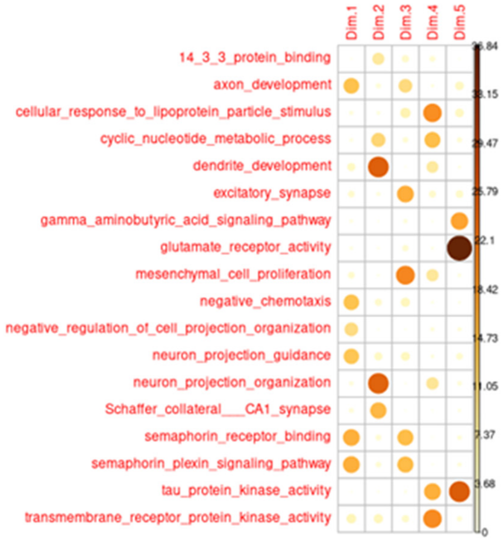

Replication

Supplementary Figure 7 Pathway Validation

Pathway validation a) Association of pathway-specific unweighted methylation risk scores (MRS) with MDD and rMDD in the discovery (circles) and replication (triangles) sets; b) polygenic risk score analysis in the replication sample with weighting from MDD – Wray *et al.* 2018, PTSD -Nievergelt *et al.* 2024, trauma exposure – Coleman *et al.* 2021, trauma exposure2 – Carey *et al.* 2024; c) pathway-specific LD score regression using published GWAS (Base): MDD - Wray *et al.* 2018, PTSD – Nievergelt *et al.* 2024, and trauma exposure – Coleman *et al.* 2021 and trauma exposure 2 – Carey *et al.* 2024 with vertical dashed line at a mean chi-square of 1.2 (mean per SNP chi-squared in the base analyses) and asterisks representing the *p*-value for comparison of the mean Chi2 of each pathway with the corresponding base set. For full results, including trauma exposure 2 – Carey *et al.* 2024, see Supplementary Figure 7. *p*-values: * < 0.05, **<0.001

A

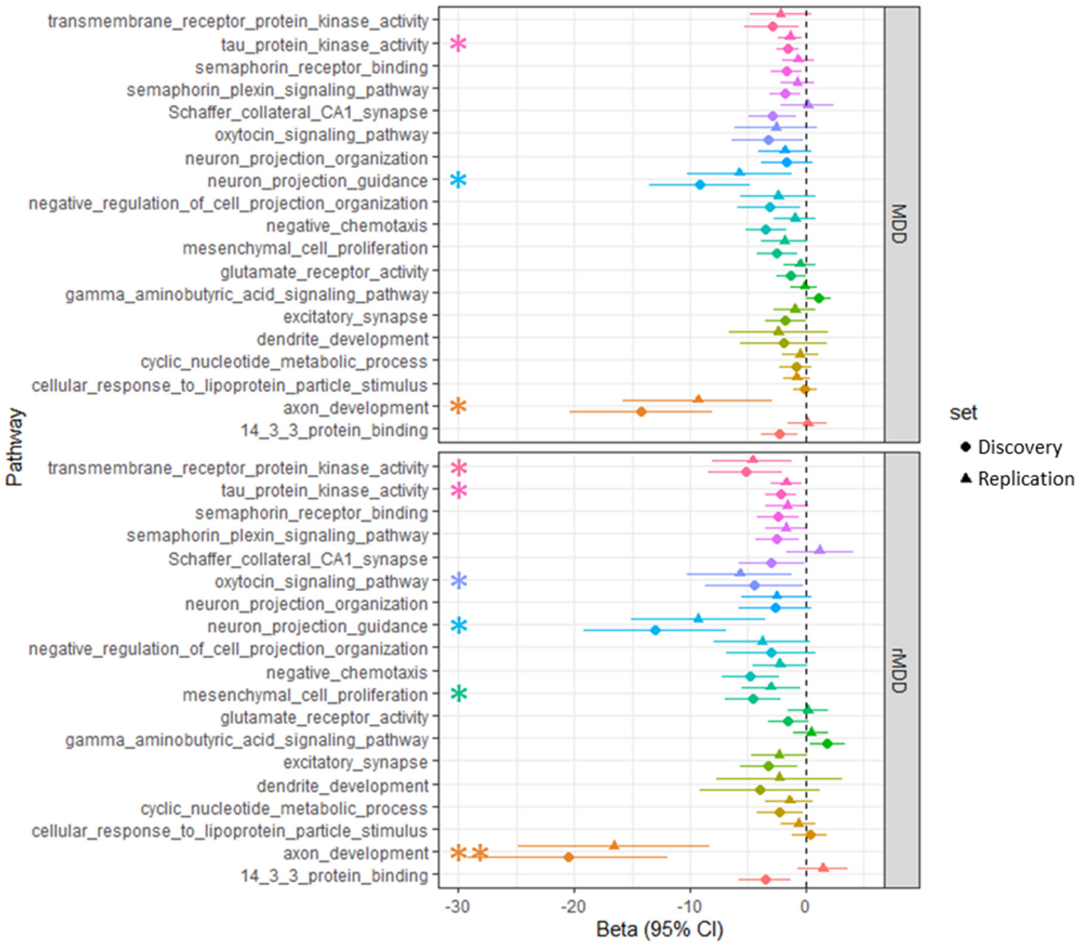

B

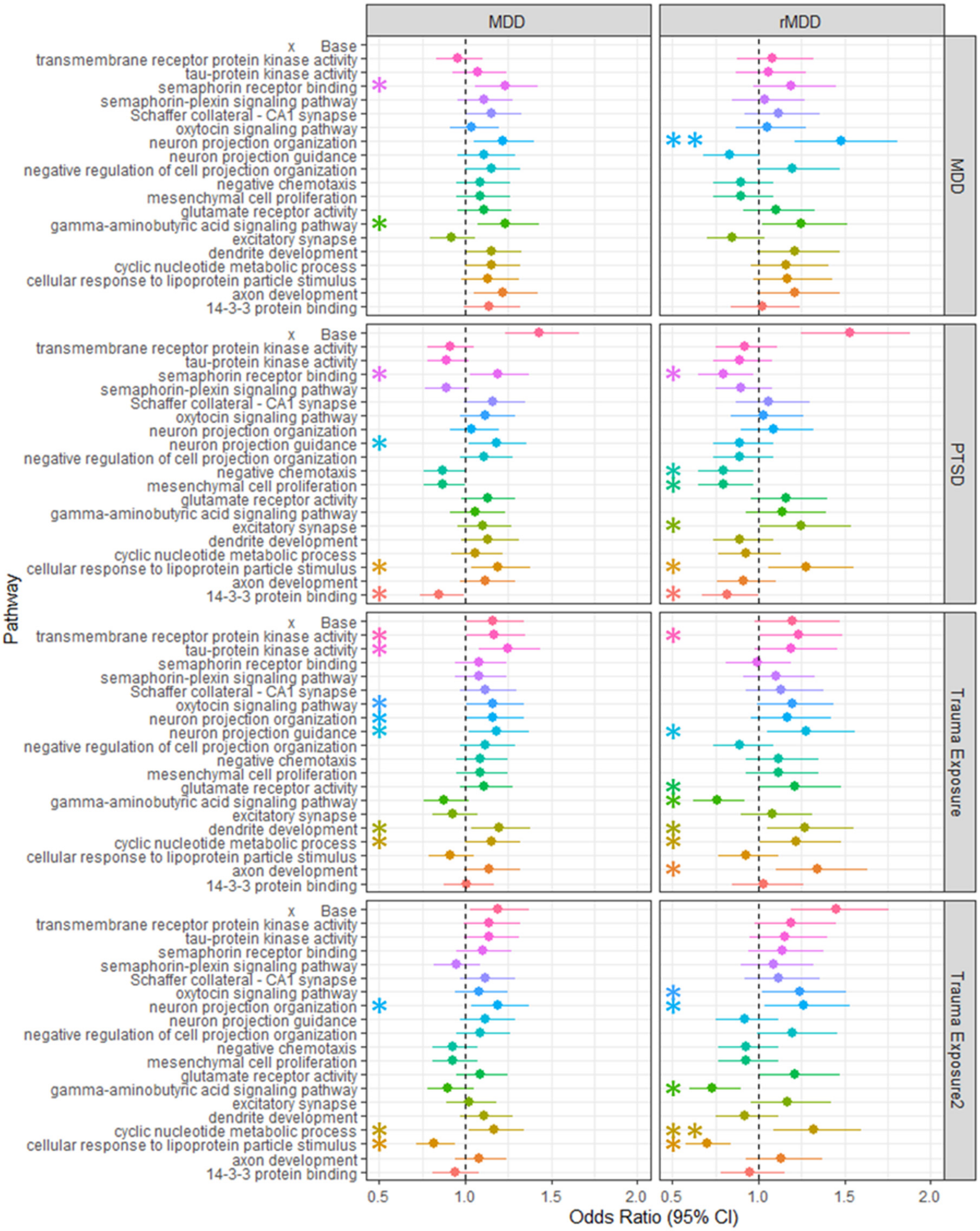

MDD
rMDD
PTSD
Trauma Exposure
Trauma Exposure2
Pathway
x Base
transmembrane receptor protein kinase activity
tau-protein kinase activity
semaphorin receptor binding
semaphorin-plexin signaling pathway
Schaffer collateral - CA1 synapse
oxytocin signaling pathway
neuron projection organization
neuron projection guidance
negative regulation of cell projection organization
negative chemotaxis
mesenchymal cell proliferation
glutamate receptor activity
gamma-aminobutyric acid signaling pathway
excitatory synapse
dendrite development
cyclic nucleotide metabolic process
cellular response to lipoprotein particle stimulus
axon development
14-3-3 protein binding
0.5
1.0
1.5
2.0
Odds Ratio (95% CI)

C

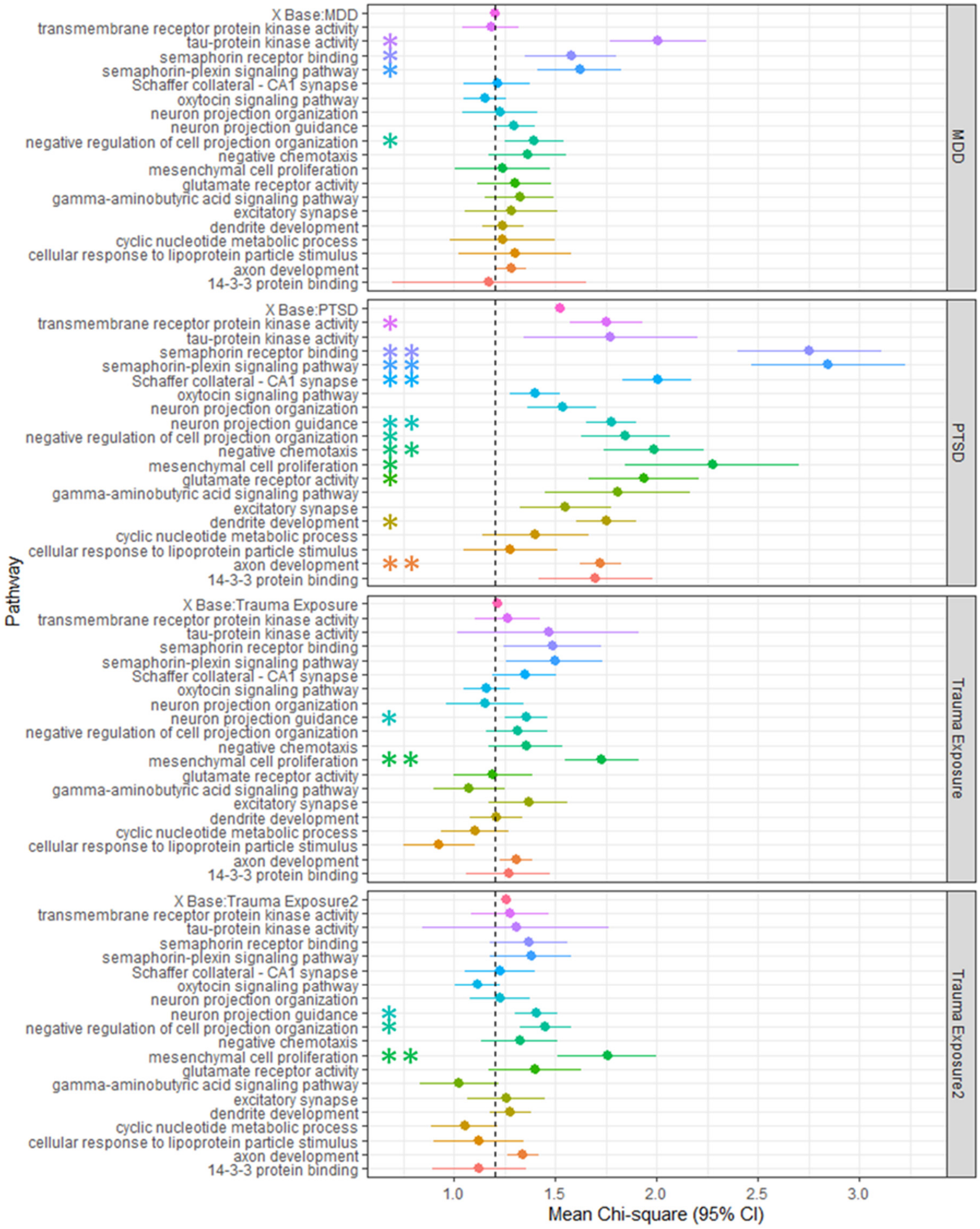

X Base:MDD
transmembrane receptor protein kinase activity
tau-protein kinase activity
semaphorin receptor binding
semaphorin-plexin signaling pathway
Schaffer collateral - CA1 synapse
oxytocin signaling pathway
neuron projection organization
neuron projection guidance
negative regulation of cell projection organization
negative chemotaxis
mesenchymal cell proliferation
glutamate receptor activity
gamma-aminobutyric acid signaling pathway
excitatory synapse
dendrite development
cyclic nucleotide metabolic process
cellular response to lipoprotein particle stimulus
axon development
14-3-3 protein binding
MDD
X Base:PTSD
PTSD
X Base:Trauma Exposure
Trauma Exposure
X Base:Trauma Exposure2
Trauma Exposure2
Pathway
1.0
1.5
2.0
2.5
3.0
Mean Chi-square (95% CI)

References

1. John, Raven J (2003): Raven Progressive Matrices. In: McCallum RS, editor. *Handbook of Nonverbal Assessment*. Boston, MA: Springer US, pp 223-237.
2. Wechsler D (1997): WAIS-III administration and scoring manual. The Psychological Corporation, San Antonio, TX. *Wechsler, D(1997) WAIS III Adm Scoring Manual Psychol Corp San Antonio, TX*.
3. Wechsler III D (1997): WMS-III Administration and Scoring Manual. San Antonio, TX: The Psychological Corporation. Harcourt Brace & Co.
4. Eysenck S, Eysenck H, Barrett P (1985): Short-scale Eysenck Personality Questionnaire--Revised. *Personality and Individual Differences*.
5. Goldberg D (1978): *Manual of the General Health Questionnaire*. NFER.
6. Hirschfeld RM, Williams JB, Spitzer RL, Calabrese JR, Flynn L, Keck PE, Jr., et al. (2000): Development and validation of a screening instrument for bipolar spectrum disorder: the Mood Disorder Questionnaire. *Am J Psychiatry*. 157:1873-1875.
7. Davidson CA, Hoffman L, Spaulding WD (2016): Schizotypal personality questionnaire--brief revised (updated): An update of norms, factor structure, and item content in a large non-clinical young adult sample. *Psychiatry Res*. 238:345-355.
8. Raine A (1991): The SPQ: a scale for the assessment of schizotypal personality based on DSM-III-R criteria. *Schizophr Bull*. 17:555-564.
9. Barbu MC, Amador C, Kwong ASF, Shen X, Adams MJ, Howard DM, et al. (2022): Complex trait methylation scores in the prediction of major depressive disorder. *EBioMedicine*. 79:104000.
10. Bermingham ML, Walker RM, Marioni RE, Morris SW, Rawlik K, Zeng Y, et al. (2019): Identification of novel differentially methylated sites with potential as clinical predictors of impaired respiratory function and COPD. *EBioMedicine*. 43:576-586.
11. Madden RA, McCartney DL, Walker RM, Hillary RF, Bermingham ML, Rawlik K, et al. (2021): Birth weight associations with DNA methylation differences in an adult population. *Epigenetics*. 16:783-796.
12. Walker RM, Vaher K, Bermingham ML, Morris SW, Bretherick AD, Zeng Y, et al. (2021): Identification of epigenome-wide DNA methylation differences between carriers of APOE epsilon4 and APOE epsilon2 alleles. *Genome Med*. 13:1.
13. Smyth G (2005): Limma: linear models for microarray data. Gentleman RCarey VDudoit SIrizarry RHuber W Bioinformatics and computational biology solutions using R and Bioconductor. New York: Springer.
14. Clogg CC, Petkova E, Haritou A (1995): Statistical Methods for Comparing Regression Coefficients Between Models. *Am J Sociol*. 100:1261-1293.
15. Mansell G, Gorrie-Stone TJ, Bao Y, Kumari M, Schalkwyk LS, Mill J, et al. (2019): Guidance for DNA methylation studies: statistical insights from the Illumina EPIC array. *BMC Genomics*. 20:366.
16. Zhou Y, Zhou B, Pache L, Chang M, Khodabakhshi AH, Tanaseichuk O, et al. (2019): Metascape provides a biologist-oriented resource for the analysis of systems-level datasets. *Nat Commun*. 10:1523.
17. Evangelista JE, Xie Z, Marino GB, Nguyen N, Clarke DJB, Ma'ayan A (2023): Enrichr-KG: bridging enrichment analysis across multiple libraries. *Nucleic Acids Res*. 51:W168-W179.
18. Mitchell DC, Kuljanin M, Li J, Van Vranken JG, Bulloch N, Schweppe DK, et al. (2023): A proteome-wide atlas of drug mechanism of action. *Nat Biotechnol*. 41:845-857.
19. Liao Y, Wang J, Jaehnig EJ, Shi Z, Zhang B (2019): WebGestalt 2019: gene set analysis toolkit with revamped UIs and APIs. *Nucleic Acids Res*. 47:W199-W205.
20. Lê S, Josse J, Husson F (2008): FactoMineR: An R Package for Multivariate Analysis. *Journal of Statistical Software*. 25:1 - 18.

21. Howard DM, Adams MJ, Clarke TK, Hafferty JD, Gibson J, Shirali M, et al. (2019): Genome-wide meta-analysis of depression identifies 102 independent variants and highlights the importance of the prefrontal brain regions. *Nat Neurosci*. 22:343-352.
22. Kerr SM, Campbell A, Murphy L, Hayward C, Jackson C, Wain LV, et al. (2013): Pedigree and genotyping quality analyses of over 10,000 DNA samples from the Generation Scotland: Scottish Family Health Study. *BMC Med Genet*. 14:38.
23. Carey CE, Shafee R, Wedow R, Elliott A, Palmer DS, Compitello J, et al. (2024): Principled distillation of UK Biobank phenotype data reveals underlying structure in human variation. *Nat Hum Behav*. 8:1599-1615.
24. Coleman JRI, Peyrot WJ, Purves KL, Davis KAS, Rayner C, Choi SW, et al. (2020): Genome-wide gene-environment analyses of major depressive disorder and reported lifetime traumatic experiences in UK Biobank. *Mol Psychiatry*. 25:1430-1446.
25. Nievergelt CM, Maihofer AX, Atkinson EG, Chen CY, Choi KW, Coleman JRI, et al. (2024): Genome-wide association analyses identify 95 risk loci and provide insights into the neurobiology of post-traumatic stress disorder. *Nat Genet*. 56:792-808.
26. Wray NR, Ripke S, Mattheisen M, Trzaskowski M, Byrne EM, Abdellaoui A, et al. (2018): Genome-wide association analyses identify 44 risk variants and refine the genetic architecture of major depression. *Nat Genet*. 50:668-681.
27. Choi SW, O'Reilly PF (2019): PRSice-2: Polygenic Risk Score software for biobank-scale data. *Gigascience*. 8.
28. Bulik-Sullivan BK, Loh PR, Finucane HK, Ripke S, Yang J, Schizophrenia Working Group of the Psychiatric Genomics C, et al. (2015): LD Score regression distinguishes confounding from polygenicity in genome-wide association studies. *Nat Genet*. 47:291-295.
29. Rai MF, Cai L, Tycksen ED, Keener J, Chamberlain A (2022): RNA-Seq reveals distinct transcriptomic differences in rotator cuff tendon based on tear etiology and patient sex. *J Orthop Res*. 40:2728-2742.
30. Hillary RF, McCartney DL, Smith HM, Bernabeu E, Gadd DA, Chybowska AD, et al. (2023): Blood-based epigenome-wide analyses of 19 common disease states: A longitudinal, population-based linked cohort study of 18,413 Scottish individuals. *PLoS Med*. 20:e1004247.
31. McCarthy S, Das S, Kretzschmar W, Delaneau O, Wood AR, Teumer A, et al. (2016): A reference panel of 64,976 haplotypes for genotype imputation. *Nat Genet*. 48:1279-1283.
